# The Formation of the Eastern Africa Rabies Network: A Sub-Regional Approach to Rabies Elimination

**DOI:** 10.3390/tropicalmed2030029

**Published:** 2017-07-18

**Authors:** Emily G. Pieracci, Terence P. Scott, Andre Coetzer, Mwatondo Athman, Arithi Mutembei, Abraham Haile Kidane, Meseret Bekele, Girma Ayalew, Samson Ntegeyibizaza, Justine Assenga, Godson Markalio, Peninah Munyua, Louis H. Nel, Jesse Blanton

**Affiliations:** 1 National Center for Emerging and Zoonotic Infectious Diseases, Centers for Disease Control and Prevention, Atlanta, GA 30329, USA; JBlanton@cdc.gov; 2 Department of Microbiology and Plant Pathology, Faculty of Natural and Agricultural Sciences, University of Pretoria, 0028 Pretoria, South Africa; andre.coetzer@rabiesalliance.org (A.C.); louis.nel@rabiesalliance.org (L.H.N.); 3 Global Alliance for Rabies Control SA NPC, Erasmus Forum A434, South Erasmus Rand, 0181 Pretoria, South Africa; 4 Zoonotic Disease Unit, Kenya Ministry of Health, Nairobi, Kenya; amwatondo@yahoo.com (M.A.); drmutembei@gmail.com (A.M.); 5 Ethiopian Public Health Institute, Addis Ababa, Ethiopia; abrahamhaile21@gmail.com; 6 Ethiopian Ministry of Livestock and Fisheries, Addis Ababa, Ethiopia; yohanan14sm@gmail.com; 7 Ethiopian Ministry of Wildlife and Conservation, Addis Ababa, Ethiopia; girmasas@gmail.com; 8 Rwandan Ministry of Agriculture, Kigali, Rwanda; ntegsam@yahoo.fr; 9 Tanzania Directorate of Veterinary Services, Ministry of Agriculture, Dar es Salam, Tanzania; assengakanda@yahoo.com; 10 Tanzania Ministry of Health, Dar es Salam, Tanzania; markalio@yahoo.com; 11 Division of Global Health Protection, Centers for Disease Control and Prevention, Nairobi, Kenya; ikg2@cdc.gov

**Keywords:** rabies surveillance, rabies network, Africa, rabies elimination

## Abstract

International rabies networks have been formed in many of the canine-rabies endemic regions around the world to create unified and directed regional approaches towards elimination. The aim of the first sub-regional Eastern Africa rabies network meeting, which included Kenya, Ethiopia, Tanzania, Rwanda, and Uganda, was to discuss how individual country strategies could be coordinated to address the unique challenges that are faced within the network. The Stepwise Approach towards Rabies Elimination and the Global Dog Rabies Elimination Pathway tool were used to stimulate discussion and planning to achieve the elimination of canine-mediated human rabies by 2030. Our analysis estimated a total dog population of 18.3 million dogs in the Eastern Africa region. The current dog vaccination coverage was estimated to be approximately 5% (915,000 dogs), with an estimated 4910 vaccinators available. Assuming that every vaccinator performs rabies vaccination, this equated to each vaccinator currently vaccinating 186 dogs per year, whilst the target would be to vaccinate 2609 dogs every year for the community to reach 70% coverage. In order to achieve the World Health Organization-recommended 70% vaccination coverage, an additional 11 million dogs need to be vaccinated each year, pointing to an average annual shortfall of $ 23 million USD in current spending to achieve elimination by 2030 across the region. Improved vaccination efficiency within the region could be achieved by improving logistics and/or incorporating multiple vaccination methods to increase vaccinator efficiency, and could serve to reduce the financial burden associated with rabies elimination. Regional approaches to rabies control are of value, as neighboring countries can share their unique challenges while, at the same time, common approaches can be developed and resource-saving strategies can be implemented.

## 1. Introduction

Rabies is a neglected disease that kills an estimated 59,000 people every year, with more than 21,000 of those deaths (36%) occurring in Africa [1,2]. As rabies is preventable, every death should be considered a public health failure. However, one human still dies from rabies every 9 minutes, with the majority of these deaths occurring in low- and middle-income countries (LMICs) in Africa and Asia [1].

In line with the Sustainable Development Goals (SDGs) [3], a preponderance of African governments seeks to emulate other continents that have drastically reduced the number of human deaths from rabies and controlled or eliminated the disease from the domestic dog, e.g., the Americas and Europe [4,5]. The vast majority of human rabies deaths are attributed to bites from rabid dogs, with children and individuals in poor communities being disproportionately affected [1,2,5,6]. Despite effective preventive measures and available post-exposure prophylaxis in humans, the most cost-effective control measure to eliminate canine-mediated human rabies remains the routine vaccination of dogs [4,7]. Across Africa, however, canine mass vaccination efforts are primarily still in the developmental stages. The global community, led by the World Health Organization (WHO), World Organisation for Animal Health (OIE), the Food and Agricultural Organization (FAO), and the Global Alliance for Rabies Control (GARC), has set a globally agreed upon goal to eliminate canine-mediated human rabies deaths by 2030 [8].

International rabies networks have been formed in many of the canine-rabies endemic regions to create unified and directed approaches towards elimination within the given regions. The Meeting of Directors of Rabies Programs in the Americas (REDIPRA) structure acted as a regional network, encompassing 27 countries in the Americas [9]. Additionally, smaller multi-national strategic planning groups were implemented to drive rabies elimination efforts; for example the North American Rabies Management Plan (NARMP) was created with a focus on wildlife rabies elimination between Canada, Mexico, and the United States [10]. In Africa, the Pan-African Rabies Control Network (PARACON), under the secretariat of GARC, was recently established as the regional network for sub-Saharan African countries [11], but smaller, community-based, sub-regional planning structures have not been implemented. For this reason, the United States Centers for Disease Control and Prevention (CDC) and GARC worked with regional leaders to establish an Eastern African rabies control planning commission. The aim of this group is to discuss how individual country strategies could be coordinated to address the unique challenges that are faced in terms of rabies control efforts within the region.

The first Eastern African regional rabies control group meeting was held from 7–9 February 2017 in Nairobi, Kenya, and was hosted collaboratively by GARC, CDC, and the Kenya Zoonotic Disease Unit (Kenya ZDU). Representatives from four Eastern African countries, along with regional and international rabies partners, attended the meeting to assess the sub-region’s current rabies control strategies, develop sub-regional targets for the future, and showcase a needs assessment analysis to approximate the cost of rabies elimination within the sub-regional network. One Eastern African country was unable to attend, but provided its data the following week. All five countries’ data were included in the model for projecting resource needs for canine rabies vaccination.

## 2. The Stepwise Approach towards Rabies Elimination

In a detailed workshop focused on the Stepwise Approach towards Rabies Elimination (SARE), and its linkage to the Rabies Blueprint (http://rabiesblueprint.org/), country representatives identified the current status of rabies control within their country. The SARE assessment provided countries with measurable steps to progress from canine rabies endemic to a canine rabies free status [12]. Representatives outlined these steps to prioritize short- and medium-term activities for each country towards a dynamic and ongoing development and implementation of national rabies control plans. Common priority activities from each of the countries were compiled into a sub-regional assessment to provide a basis for the development of a comprehensive sub-regional roadmap for the network. The development of this roadmap is focused on facilitating partnerships and coordination between countries in an effort to address the transboundary nature of canine rabies.

## 3. Regional Business Plan and Estimated Needs for Rabies Mass Vaccination

Country representatives used the Global Dog Rabies Elimination Pathway (GDREP) model for projecting resource needs for canine rabies vaccination (e.g., vaccines, vaccinator resources, and funds) [13]. The GDREP model accounts for resources needed to achieve elimination of canine-mediated human rabies, accommodating varied inputs for cost of dog vaccination, availability of vaccine, and existing workforce available for vaccinating animals to generate outputs of estimated resources needed to achieve vaccination goals. The analysis estimated a total dog population of 18.3 million dogs, based upon the Human to Dog Ratio (HDR) method [6,14,15,16], for the region (Kenya, Ethiopia, Tanzania, Rwanda, and Uganda). The current dog vaccination coverage in the region was estimated to be approximately 5% (915,000 dogs), with an estimated 4910 vaccinators available to be mobilized for vaccination campaigns [1]. This is likely to be sufficient personnel to achieve 70% vaccination of dog populations in the region, based on projections made by the GDREP tool. The estimated cost of the current 2017 level of 5% vaccination coverage (915,000 dogs) was approximately $ 3.2 million USD per year, equating to an average of $ 3.50 USD per vaccinated dog. In order to achieve the recommended 70% vaccination coverage necessary to eliminate canine rabies [5], an additional 11 million dogs will need to be vaccinated each year, equating to an approximately $ 23 million USD annual shortfall in current spending across the region.

The total cost of vaccinating a single dog encompasses supplies and personnel, not just the cost of the vaccine alone. Improving the efficiency of vaccination programs by improving logistics and/or incorporating multiple vaccination methods (e.g., central point vaccination, capture-vaccinate-release), could reduce overall costs [13]. Considering the range of vaccination costs reported in the GDREP [13], from $ 1.50/dog to $ 7.00/dog, the estimated average annual shortfall in current spending is likely between $ 8.5 million USD and $ 49.8 million USD. Improvements in the planning and efficiency of national canine rabies vaccination campaigns coupled with frequent refinements of national strategies will be critical to ensure that the expected shortfall is reduced and additional funds can be secured. Sharing of resources and technical experience between regional countries will help to facilitate these improvements and more efficiently identify and secure support from national and international partners.

## 4. Data Sharing Within the Eastern African Rabies Region

The PARACON epidemiological bulletin, a web-based surveillance database, is a useful tool for the collection, collation, analysis, and dissemination of rabies surveillance data [17]. To encourage the use of the bulletin, sub-regional country-specific data were collected from meeting participants, and collated data were summarized on a single viewable page. This ‘Eastern Africa Dashboard’ was made available to all of the participating countries within the network and is envisaged to encourage international collaboration and communication. With increased transparency, a stronger case can be made for transboundary control effort by the participating countries, drawing global interest and raising awareness and advocacy for rabies control within the region. It is also important to engage other Eastern African countries (Burundi and South Sudan) in the Eastern Africa rabies network to achieve a unified regional approach.

## 5. Conclusions

Rabies prevention should be considered a free public good globally. Despite the presence of an effective vaccine for the prevention of rabies for more than a hundred years, rabies continues to kill thousands of people across the globe each year [1,18]. Some countries have achieved success in eliminating rabies through effective, organized, and coordinated control efforts, but many LMICs remain rabies endemic.

Across Africa, rabies control and elimination efforts are primarily still in the developmental stages. A concerted and carefully conceived approach will need to be created and undertaken to ensure that the goal of elimination by 2030 is reached. The overarching PARACON network guides African countries and provides exposure to new and innovative tools and approaches, while facilitating the development and revision of their national control strategies [11]. Although each country within the PARACON network has access to the same tools, the implementation and use of these tools will often need to be adapted to sub-regions that experience different or additional challenges to those faced by Africa as a whole. Thus, the establishment of the Eastern Africa rabies network has addressed this issue by providing a close-knit working group within the PARACON network for strategic planning and cooperation.

These tools, in conjunction with the increased transparency between the participating countries, and leveraging on existing regional economic and technical bodies promoting the One Health approach, will enable this network to develop their own sustainable roadmap towards rabies control and elimination, with a focus on addressing the transboundary nature of the disease throughout the region. It is envisaged that this roadmap will provide detailed priority activities, steps, and plans directed to the mobilization and unification of the community towards the common goal of canine-mediated human rabies elimination. For the Eastern Africa rabies network, Kenya was nominated to act as the chair on a two-year rotational basis and will be the leading country in the development of such a roadmap for presentation, review, and discussion at the next PARACON meeting (2017). By sharing the roadmap and presenting the concept of the smaller focused communities within the PARACON network, the Eastern Africa rabies network will act as a flagship for other communities with common challenges to unite and tailor the foundational basis of the roadmap to their specific needs.
